# Dissecting the components of error in analogue report tasks

**DOI:** 10.3758/s13428-024-02453-w

**Published:** 2024-07-08

**Authors:** Ivan Tomić, Dagmar Adamcová, Máté Fehér, Paul M. Bays

**Affiliations:** 1 Department of Psychology, University of Cambridge, Cambridge, England; 2 Department of Psychology, Faculty of Humanities and Social Sciences, University of Zagreb, Ivana Lucica 3, 10000 Zagreb, Croatia; 3 Faculty of Biology, University of Cambridge, Cambridge, England

**Keywords:** Analogue report task, Fidelity, Response variability, Motor noise, Adjustment criterion

## Abstract

Over the last two decades, the analogue report task has become a standard method for measuring the fidelity of visual representations across research domains including perception, attention, and memory. Despite its widespread use, there has been no methodical investigation of the different task parameters that might contribute to response variability. To address this gap, we conducted two experiments manipulating components of a typical analogue report test of memory for colour hue. We found that human response errors were independently affected by changes in storage and maintenance requirements of the task, demonstrated by a strong effect of set size even in the absence of a memory delay. In contrast, response variability remained unaffected by physical size of the colour wheel, implying negligible contribution of motor noise to task performance, or by its chroma radius, highlighting non-uniformity of the standard colour space. Comparing analogue report to a matched forced-choice task, we found variation in adjustment criterion made a limited contribution to analogue report variability, becoming meaningful only with low representational noise. Our findings validate the analogue report task as a robust measure of representational fidelity for most purposes, while also quantifying non-representational sources of noise that would limit its reliability in specialized settings.

**Supplementary Information:**

The online version contains supplementary material available at 10.3758/s13428-024-02453-w.

## Dissecting the components of error in analogue report tasks

The analogue report task (Prinzmetal et al., 1998; Wilken & Ma, 2004) provides a method of measuring the fidelity with which humans (and other animals) internally represent visual information. Typically, the task consists of presenting one or more stimuli that vary pseudorandomly with respect to a low-level visual feature dimension. The continuum of features from which stimuli are selected is commonly chosen to have the topology of a circle, as e.g. planar orientation, colour hue, or motion direction. At test, a single target stimulus is indicated by a secondary feature (e.g. its location), and observers are asked to identify the specific report feature value of that stimulus by selection from the full feature space (e.g. by clicking on a colour wheel). Repeating such a procedure across many trials with random stimulus feature values results in a distribution of response errors that is approximately symmetric and bell-shaped, and the width of this distribution can be interpreted as a measure of the fidelity of internal representations. It has become common practice to further decompose these response distributions using mixture models, which attempt to statistically distinguish errors attributable to noisy representations of the target, lapses producing random guesses (Zhang & Luck, 2008) and intrusions of non-target features (swap errors) (Bays et al., 2009; McMaster et al., 2022). The ability to quantitatively reproduce patterns of error on analogue report tasks is a widely used criterion for selecting between competing computational or neural models of internal representation.

Over recent years, studies using variations of this task have shaped our understanding of cognitive functions including visual perception (Bays, 2016; Thibault et al., 2016), attention (Murray et al., 2013; Tang et al., 2020), sensory memory (Pratte, 2018; Tomić & Bays, 2024a), working memory (Bays et al., 2009; Fougnie et al., 2012; Schurgin et al., 2020; van den Berg et al., 2012; Zhang & Luck, 2008), and long-term memory (Brady et al., 2013; Richter et al., 2016). Despite its prevalence, a comprehensive study of factors contributing to responses on this task is still lacking.

Responses on the analogue report task are by nature variable, i.e. asking an observer to reproduce the same, clearly visible stimulus multiple times will not result in identical responses, but instead produce a distribution of responses in the vicinity of the stimulus’ true feature value. One component of this variability is internal noise arising from the inherently stochastic process of mapping the external stimulus to an internal representation (Faisal et al., 2008; Tolhurst et al., 1983). While internal noise sets an upper bound on the attainable fidelity, its effects can be amplified by manipulations that decrease signal amplitude. In research on working memory, this can be achieved by increasing the number of presented stimuli, with numerous studies demonstrating a monotonic increase in response error variability with set size (Bays et al., 2009; Ma et al., 2014; Tomić & Bays, 2024a; van den Berg et al., 2012). Similarly, studies on sensory and working memory have shown that, in addition to noisy encoding, the maintenance of information in the absence of direct perception introduces additional error in feature reproduction, with the effect becoming increasingly pronounced with retention time (Pratte, 2018; Schneegans & Bays, 2018; Shin et al., 2017).

In otherwise identical psychophysical tasks, performance is known to vary depending on the stimulus feature being used (e.g. colour vs. angular location; Tomić & Bays, 2024b) and in some cases within a stimulus feature depending on the exact type of visual objects being used (e.g. oriented Gabors vs. lines; Alvarez & Cavanagh, 2008), or some secondary characteristic of the stimulus (e.g. the contrast of oriented Gabors; Bays, 2016; Tomić & Bays, 2018). These observations emphasize the importance of carefully selecting a stimulus feature space appropriate for the specific purpose of testing. In recent years, most applications of the analogue report task have used colour hue, defined as a circle of constant luminance within CIE Lab space, as the stimulus feature. This decision is strongly influenced by arguments regarding the perceptual uniformity of CIE Lab space, within which equal distances between colours are believed to correspond to equal perceptual discriminability. Selecting a circle of hues in CIE Lab space is then expected to provide a perceptually homogeneous space. On this basis, when hues are selected from a circle with a reduced radius, they should become less perceptually discriminable, leading to larger angular errors in the analogue report task. However, the impact of specific choices related to the colour space, such as chroma radius, on reproduction performance in this task has not yet been empirically investigated.

Estimates of fidelity obtained in the analogue report task are based on indirect measurements of representational quality. Commonly, observers are asked to pick a target feature from a continuous space by, e.g. clicking on a colour wheel or scrolling through the response space using keyboard keys or a response dial. In such cases, the selection of a specific feature can be contaminated by motor noise, due to which the internally selected feature is imperfectly translated to a hand movement, resulting in increased variability of response errors. This source of error has been acknowledged by and built into computational models of visual processing (Schurgin et al., 2020; van den Berg et al., 2012), however we are not aware of any previous attempt to directly quantify the contribution of motor noise to overall reproduction variability.

Historically, in visual psychophysics, the analogue report task and its predecessor method of adjustment were used less often than forced-choice methods (Green & Swets, 1966; Kingdom & Prins, 2016). One particular reason for that is that in the continuous report methods, observers have the freedom to decide when to terminate the adjustment process (i.e. how similar is “similar enough”), and consequently, this choice will reflect in the overall distribution of responses. In particular, an observer with a very liberal criterion when identifying the target feature, or an unstable criterion that varies during the task, will produce more variable responses compared to an observer with the same ability but a more conservative and stable adjustment criterion. This is different from a typical forced-choice method in which observers are asked to correctly choose between multiple (typically two) alternatives, while the experimenter determines the limit to the observers’ accuracy by defining the similarity of the alternatives. Nevertheless, except for early comparisons of continuous adjustment and forced-choice methods in the domain of auditory absolute and difference thresholds (Wier et al., 1976; Cardozo, 1965), there is little evidence as to whether the adjustment criterion presents a substantial source of variability in analogue report.

In the present study, we investigated factors arising both from limitations of the information processing system and choices related to the task that might contribute to the overall variability of report errors and affect estimates of fidelity in the analogue report task. In two experiments, we used the analogue report task with colour hue as report feature to systematically investigate influences of set size, delay, chroma radius and physical size of the colour wheel on human reproduction fidelity under both perceptual and working memory conditions, and compared results with performance of the same participants on a matched forced-choice task.

**Fig. 1:**
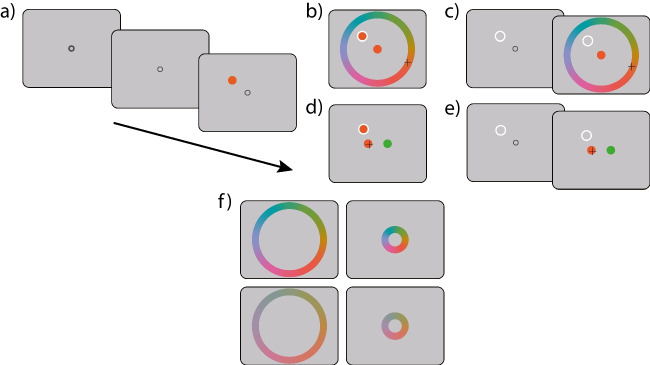
Experimental tasks. *Note.* **(a)** Each trial of the study began with the presentation of a fixation marker followed by a stimulus array consisting of one or four coloured discs. **(b)** In the synchronous report condition of the analogue report task, a single presented item remained visible on the screen during the response, and observers were asked to identify its colour by clicking with a crosshairs cursor on the colour wheel. The currently selected colour was presented in a central disc during the response. **(c)** In the asynchronous report condition of the analogue report task, the stimulus array disappeared, and one item was cued by displaying a ring at the item’s location. In the no-delay experiment, the cue followed the stimulus array immediately, and in the delay experiment, the array and cue were separated by a 1-s blank display (not shown here). **(d)** In the synchronous report 2-AFC task, two coloured discs were presented at test along with the target stimulus, and observers were required to click on the disc that matched the target hue. **(e)** In the asynchronous report 2-AFC task, one item was cued via its location, and observers were again asked to choose which of the two presented discs matches the cued item’s hue. **(f)** Illustration of colour wheels with large (*top*) and small (*bottom*) chroma radii, and large (*left*) and small (*right*) physical sizes. Feedback displays are omitted. Displays are not to scale

## Methods

### Summary of experimental manipulations

In two experiments, two response methods were used to collect observers’ responses (Fig. 1). An analogue report task was used to collect continuous reproduction of the target colour, and a 2-AFC task was used to collect binary choices on the similarity of the target colour and two test colours. With both response methods (i.e. in the analogue report and 2-AFC task), the chroma (or ‘colourfulness’) of stimuli was manipulated such that half the trials used a colour wheel with a small chroma radius (r = 25), and the other half used a colour wheel with a large chroma radius (r = 50). On an individual trial, the same colour wheel was used for sampling the study array colours and in test arrays for sampling foils and defining response wheels (Fig. 1f).

With both response methods, the test cues were presented and observers provided responses synchronously or asynchronously with respect to the study array. Specifically, on synchronous trials, one coloured disc was presented simultaneously with the test cue and observers were asked to provide a response while the stimulus was visible on the screen. On asynchronous trials, one or four coloured discs were displayed, and following their offset, the test cue was presented indicating the location of the item to be recalled. With the asynchronous responses, we had two delay conditions: no delay (i.e. 0-ms delay in Experiment 1; the no-delay experiment) and delay (i.e. 1000 ms in Experiment 2; the delay experiment). The choice of a 1-s delay in the delay experiment was guided by its typical use in VWM studies (research examining the effects of different delay durations on analogue recall performance includes (Rademaker et al., 2018; Schneegans & Bays, 2018; Shin et al., 2017; Tomić & Bays, 2024a).

In the no-delay experiment, in the response phase of the analogue report task, we manipulated the physical size of the colour wheel used for responding, such that observers gave their responses on a physically small (radius $3^{\circ }$) or large colour wheel (radius $10^{\circ }$). While altering the chroma radius affected the colourfulness of stimuli and response wheels, altering the physical size of the response wheel did not affect its colour, making these manipulations conceptually orthogonal (Fig. 1f).

### Participants

A total of 20 naive observers (ten females, nine males, one non-binary, age 18–39) took part in the study after giving informed consent in accordance with the Declaration of Helsinki. The sample size was determined based on typical sample sizes used in studies involving analogous report tasks (e.g. Bays, 2014; van den Berg et al., 2012; Zhang & Luck, 2008). All observers reported normal or corrected-to-normal visual acuity and normal colour vision, and were remunerated £10 per hour for their participation. Procedures were approved by the University of Cambridge Psychology Research Ethics Committee. Ten observers took part in Experiment 1 (no-delay experiment), and the remaining ten observers took part in Experiment 2 (delay experiment).

### Stimuli and apparatus

Stimuli were presented on a 69-cm gamma-corrected LCD monitor (resolution $2560 \times 1440$) with a refresh rate of 144 Hz. Observers were seated in a dark room and viewed the monitor at a distance of 60 cm, with their head stabilized by a forehead and chin rest. Stimulus presentation and response registration were controlled using MATLAB (The MathWorks, Inc.) with the Psychophysics Toolbox extensions (Brainard, 1997; Pelli, 1997). Gaze direction was monitored online at 1000 Hz using an infrared eye tracker (SR Research). Observers were required to fixate the central fixation point from the beginning of the trial until one stimulus was cued for reproduction. Any trial on which gaze deviated $>2\deg$ from the central dot, prior to the cue, was aborted and restarted with a new set of stimuli.

Study stimuli consisted of coloured discs (size $1^{\circ }$ radius) randomly positioned at one of eight equidistant locations around the circumference of an imaginary circle ($6^{\circ }$ radius) centred on a fixation marker (Fig. 1). Disc colours were sampled randomly and independently from a colour wheel defined as a circle in CIE LAB space of constant luminance (*L*= 60), centred at *a* = *b* = 12.5, with a radius of either 25 (small chroma radius) or 50 (large chroma radius) units. All stimuli were presented against a background matched in colour to the centre of the colour wheel, but with lower luminance (*L* = 30).

### Procedure

Each experiment was divided into two sessions conducted on different days, with one session using a colour wheel with a small chroma radius and the other session using a colour wheel with a large chroma radius. The order of the sessions was counterbalanced across observers. Within each session, observers completed nine (Experiment 1, no delay) or six (Experiment 2, delay) blocks of trials in a pseudorandom order, with half of the observers completing the analogue report task followed by the 2-AFC task, and the other half completing the two tasks in the opposite order. In the no-delay experiment, observers completed a total of twelve blocks of the analogue report task and six blocks of the 2-AFC task. The larger number of blocks of the former task is due to the manipulation of the physical size of the colour wheel (see below), which was applicable only to that task. In the delay experiment, observers completed six blocks of each task.

Each trial of the study began with the presentation of a central fixation annulus (r $= 0.15^{\circ }$ and R $= 0.25^{\circ }$) (Fig. 1). Once a stable fixation was registered, the size of the inner radius increased (r $= 0.2^{\circ }$). Observers perceived this change as the annulus becoming thinner. After 500 ms, coloured discs sampled from a colour wheel with a large or small chroma radius were presented.

#### Analogue report task

In the synchronous timing condition (Fig. 1a & b), the test cue (white annulus, $1^{\circ }$ radius) was presented at the location of and along one coloured disc, and all stimuli remained visible until the end of the trial. In the asynchronous timing condition (Fig. 1a & c), one or four coloured discs were presented for 500 ms. This was followed by a 0-ms delay (no-delay experiment), or 1000-ms delay (delay experiment) and a probe display consisting of a white annulus indicating one of the stimuli to be reproduced. Participants were instructed to move the mouse once they were ready to respond. The response wheel and a central disc ($1^{\circ }$ radius) were presented on the screen only after detecting mouse movement to prevent interference from colours on the screen. The response wheel was randomly rotated from trial to trial. In the no-delay experiment, in separate blocks the response wheel was either physically small ($3^{\circ }$ radius) or large ($10^{\circ }$ radius). In the delay experiment, only the physically large ($10^{\circ }$ radius) response wheel was used. As observers hovered over the response wheel with the mouse pointer, the central disc continuously changed colour. The response was finalized with a mouse click. At the end of each trial, observers were presented with feedback in the form of the correct colour ($\textit{t}$) at the target’s original location and a central disc in the reported colour ($\textit{y}$).

Using the analogue report task, we manipulated the colour wheel’s chroma radius (small or large), the colour wheel’s physical size (small or large), the timing condition (synchronous or asynchronous response), set size (1 or 4), and delay (0 ms or 1000 ms). The physical size manipulation of the colour wheel produced no discernible effects in the no-delay experiment, and consequently, it was omitted from the delay experiment for practical considerations. Observers performed 50 trials in each condition. Trials for every condition were blocked.

#### Two-alternative forced choice task

In the 2-AFC task (Fig. 1d & e), the trial sequence was identical to the analogue report task, except for the response phase. After the probe display, participants were instructed to move the mouse when ready to respond. The probe remained on the screen until mouse movement was detected, at which point observers were presented with two coloured discs, the probe colour ($\textit{p}$; $\textit{p} = \textit{t}$) and a foil ($\textit{f}$), located $2.5^{\circ }$ horizontally to the left and right from the centre of the screen. Positions of the probe and foil were randomized on every trial. Observers gave their responses by clicking on the disc which they believed matched the colour previously presented at the cued location. At the end of each trial, observers were presented with binary feedback (“Correct” or “Incorrect”) displayed at the centre of the screen.

The colour of the foil ($\textit{f} = \textit{t} + \delta$) was selected in each trial via the adaptive PSI method to maximize the information available for estimating the discrimination function with respect to the stimulus space (Kontsevich & Tyler, 1999). The adaptive PSI method is a Bayesian method designed to estimate parameters of the psychometric function, i.e. slope and threshold. On each trial, following the observer’s response, the method updates posterior probability distributions in the parameter space and estimates parameters by calculating the means from those posteriors.

The method then selects a stimulus value (i.e. $\delta$) to present on the next trial from a dense grid across the entire feature space. The goal is to choose a value that minimizes the expected uncertainty of estimated parameters from the posterior distributions, by estimating the expected entropy across the entire feature space and choosing a stimulus value with the minimum expected entropy. The method runs for a specified number of trials, which was 80 in our case. It is important to note that while the PSI method provides online estimates of the psychometric function parameters during the experiment, the reported analyses are based on retrospectively fitting the complete set of stimuli and responses, independently of the PSI method

We again manipulated the colour wheel’s chroma radius (small or large), the timing condition (synchronous or asynchronous response), set size (1 or 4), and delay (0 ms or 1000 ms). Observers performed 80 2-AFC trials per condition, and all trials were blocked.

## Analysis

In the analogue report task, we measured response error in each trial as the angular difference between the reported ($\textit{y}$) and target ($\textit{t}$) colours on the colour wheel. To quantify the dispersion of response errors, we calculated the mean cosine dissimilarity (i.e. the complement to 1 of the cosine of response error: $1-\cos [y-t]$) across trials for each condition and observer. Higher values of cosine dissimilarity indicate larger average reproduction error.

We additionally fit a contamination model to the response distributions from each condition (code available at https://bayslab.com/toolbox/). This model assumes a probabilistic mixture of target-related responses, drawn from a von Mises (circular normal) distribution centred on the target value with concentration $\mathrm {\kappa _{t}}$, and contaminant responses that are randomly (uniformly) distributed with respect to the target, i.e.

1 $$\begin{aligned} p(y) = \alpha \phi _\textrm{VM}(y; t, \kappa _{t}) + \frac{1-\alpha }{2\pi }. \end{aligned}$$

where $\alpha$ is the mixture proportion of target-related responses. The contamination model is used here as a purely descriptive account of the data for the purpose of comparing experimental methods, without associating the two components of the model with specific psychological processes (Taylor & Bays, 2018). For the analogue report task, we analysed both the cosine dissimilarity and the parameters of the contamination model.

In the 2-AFC task we measured performance as the proportion of correct selections of the probe colour (*p*) over the foil (*f*). To facilitate the comparison with the analogue report task, we fit an instantiation of the same contamination model to the raw responses from the asynchronous condition of the 2-AFC task. We assumed that observers selected whichever of the probe and foil colours fell closest to an internal estimate (*y*) of the target colour (*t*), with *y* distributed as in Eq. 1. On this basis, the probability of a correct response as a function of target-foil similarity is given by,

2 $$\begin{aligned} \mathrm {Pr_{corr}} = \alpha \left[ \Phi _\textrm{VM}\left( \frac{|f \ominus t |}{2}; 0, \kappa _{t}\right) - \Phi _\textrm{VM}\left( \frac{|f \ominus t |}{2} - \pi ; 0, \kappa _{t}\right) \right] + \frac{1-\alpha }{2} \end{aligned}$$

where $\Phi _\textrm{VM}(\theta ; \mu , \kappa ) = \int _{-\pi }^\theta \phi _\textrm{VM}(x; \mu , \kappa )\; dx$ is the cumulative von Mises distribution, $\ominus$ is subtraction on the circle, $\mathrm {\kappa _{t}}$ is the concentration parameter and $\mathrm {\alpha }$ is the mixture proportion. Importantly, fitting the contamination model to the analogue report and forced-choice data produces directly comparable parameters: the concentration parameter indicates the level of representational precision, while lapse errors in both tasks reflect responses unrelated to the target colour.

The above model suffices for the asynchronous timing condition in both tasks, assuming that noise in the representation of stimuli that are visible at the time of response is negligible in comparison to noise in the representation of the target colour held in memory. For the synchronous timing condition, by contrast, all stimuli are visible at the time of response, so we assume that the representational noise associated with the target ($\sigma _{t}^2$), probe ($\sigma _{p}^2$), foil ($\sigma _{f}^2$) and individual colours on the colour wheel ($\sigma _{w}^2$) are small, independent and equal, and that lapses do not occur. On this basis, responses in the synchronous timing condition of the analogue report task are normally distribution,

3 $$\begin{aligned} p(y) = \phi (y;t,\sigma _{y}) \end{aligned}$$

where $\sigma _{y}$ encompasses noise in representing the target colour and the colours on the colour wheel,

4 $$\begin{aligned} \sigma _{y} = \sqrt{\sigma _{w}^2+\sigma _{t}^2} = \sqrt{2}\sigma _{t}. \end{aligned}$$

We therefore simply estimated standard deviation $\sigma _{y}$ from the raw response errors on the colour wheel.

For the synchronous timing condition of the 2-AFC task, we assumed choices reflected the same comparison between the probe, foil and target as in the asynchronous model, but with all three representations now subject to equal and independent Gaussian noise, i.e.

5 $$\begin{aligned} \mathrm {Pr_{corr}}&= \textrm{Pr}(|\hat{p} \ominus \hat{t} |< |\hat{f} \ominus \hat{t} |) \end{aligned}$$

6 $$\begin{aligned}&= \Phi _{\mu ,\Sigma }^2(0) + \Phi _{-\mu ,\Sigma }^2(0) \end{aligned}$$

where $\Phi ^2$ is the cdf of a bivariate normal with vector of means,

7 $$\begin{aligned} \mu = \left[ \frac{|f \ominus t |}{\sqrt{2}}, \frac{|f \ominus t |}{\sqrt{2}}\right] , \end{aligned}$$

and covariance matrix,

8 $$\begin{aligned} \Sigma = \begin{bmatrix} 3\sigma _t^2 & 0 \\ 0 & \sigma _t^2 \end{bmatrix}. \end{aligned}$$

Note that the asymmetry of the covariance matrix reflects the correlation induced by terms on both sides of the comparison in Eq. 5 depending on $\hat{t}$. We estimated $\sigma _t$ by maximum likelihood fitting.

Finally, we reparametrized the fitted standard deviation parameters $\sigma _t$ obtained from the synchronous tasks into concentration parameters for comparison with $\kappa _{t}$ obtained in the asynchronous tasks. In summary, when comparing performance between the analogue report and 2-AFC task, we focus on concentrations of the target-related responses, estimated either as a component of the contamination model (asynchronous report condition) or width of a Gaussian distribution (synchronous report condition). It is worth noting that when comparing performance within the analogue report task, we used the contamination model parameters for both the synchronous and asynchronous conditions.

Although fitting a parametric model to the data is only strictly necessary for the comparison between 2-AFC and analogue report tasks, considering the widespread use of such models in the field, we present contamination model parameter estimates alongside the non-parametric performance measure (cosine dissimilarity) throughout the Results section. To foreshadow our results, these parametric and non-parametric measures provided highly consistent results, underscoring the robustness and independence of our conclusions from the specific metric used to quantify representational precision.

To compare differences in performance across conditions, we used the Bayesian approach implemented in JASP (JASP Team, 2022) with the default Jeffreys–Zellner–Siow prior on effect sizes (Liang et al., 2008). The reported Bayes factors compare the predictive adequacy of two competing hypotheses (e.g. alternative and null) and quantify the change in belief that the data bring about for the hypotheses under consideration (Wagenmakers et al., 2018). For example, $\textrm{BF}_{10} = 5$ indicates that the data are five times more likely to occur under the alternative hypothesis (i.e. there is a difference) than under the null hypothesis (i.e. there is no difference). Evidence for the null hypothesis is indicated by $\textrm{BF}_{10} < 1$, in which case the strength of evidence is indicated by $1/\textrm{BF}_{10}$. Evidence assessed via the Bayes factor is most effectively understood as a ratio-scaled value ranging from 0 to infinity. Nonetheless, for the sake of clarity in communication, we also adopt an interpretative framework for Bayes factor values, following the classification scheme outlined by Lee and Wagenmakers (2013). It is critical to note that while we utilize these discrete categories, they are arbitrary and should serve only as rough guidelines. While theoretically both equally important, strong evidence, as measured by the Bayes factor, for the null hypothesis can be harder to attain in practice compared to the alternative hypothesis. This is because the predictions under the null hypothesis overlap with predictions for small effect sizes under the alternative hypothesis, leading to an asymmetry in how evidence for the null (i.e. absence of difference) and the alternative (i.e. difference) accumulate (Fig. S1) (Keysers et al., 2020; Stefan et al., 2019).

When reporting a specific effect, e.g. the effect of set size on cosine dissimilarity, we report $\textrm{BF}_{incl}$ for the factor of set size obtained from the Bayesian analysis of variance including the effects of all possible factors tested in the experiments and their interactions. For example, in the no-delay experiment, in addition to manipulating the set size of WM, we manipulated the chroma radius and the physical size of the colour wheel. When evaluating the effect of each of these three manipulations, we retrieve these effects from a single ANOVA and report $\textrm{BF}_{incl}$, which represents the overall evidence for an effect. This is derived via Bayesian model averaging (Hinne et al., 2020), which averages evidence for an effect over all candidate models that contain the effect of interest.

To estimate the contribution of motor error to response variability in the analogue report task, we assume variance in angular error on the colour wheel can be decomposed into additive motor and non-motor components, and that non-motor error is constant in angular space across changes of the physical radius of the wheel, while motor error is constant with respect to the hand/mouse movement, which maps linearly to cursor movements on the display measured in degrees of visual angle (dva). Variability in the Cartesian space of the display is related to variability in angular space for a colour wheel with physical radius *R* by

9 $$\begin{aligned} \sigma _\text {dva} \approx R\, \sigma _\text {ang}, \end{aligned}$$

where this approximation is valid as long as errors are small relative to the circumference of the wheel. Based on these assumptions we predict a difference in angular error variance on physically small and large colour wheels of

10 $$\begin{aligned} \sigma ^2_\text {small} - \sigma ^2_\text {large} = \left( \frac{\sigma _\text {motor}}{R_\text {small}}\right) ^2 - \left( \frac{\sigma _\text {motor}}{R_\text {large}}\right) ^2. \end{aligned}$$

So motor variance can be estimated as

11 $$\begin{aligned} \sigma ^2_\text {motor} = \frac{\sigma ^2_\text {small} - \sigma ^2_\text {large}}{R_\text {small}^{-2} - R_\text {large}^{-2}}, \end{aligned}$$

and motor SD (in dva) calculated as the square root of the estimated variance.

## Results

**Fig. 2:**
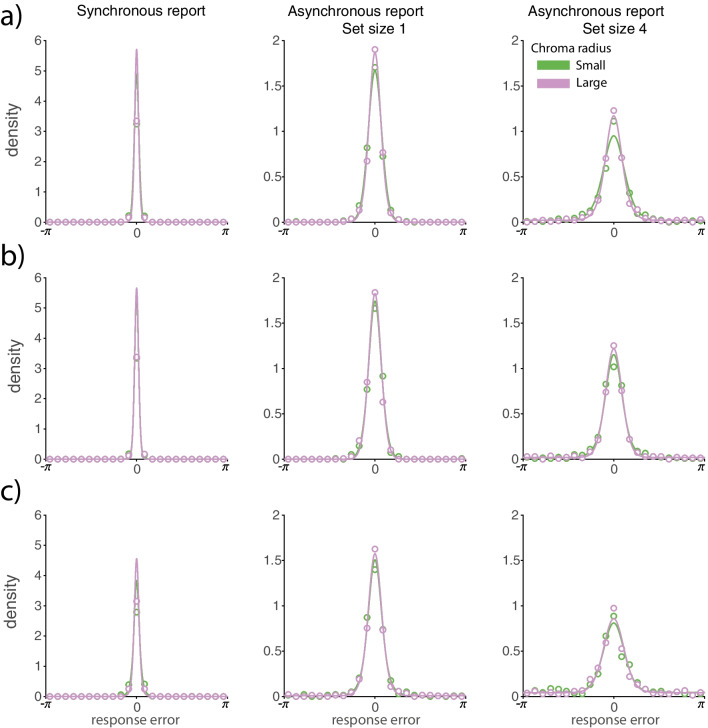
Distributions of response error in the analogue report task. *Note.* *Coloured circles* show binned response error data, and *solid lines* show the mean contamination model fit across observers. Note that the fitting procedure used raw response data and not the binned data shown here. **(a)** Distributions from the no-delay experiment with the physically small colour wheel. **(b)** Distributions from the no-delay experiment with the physically large colour wheel. **(c)** Distributions from the delay experiment

**Fig. 3:**
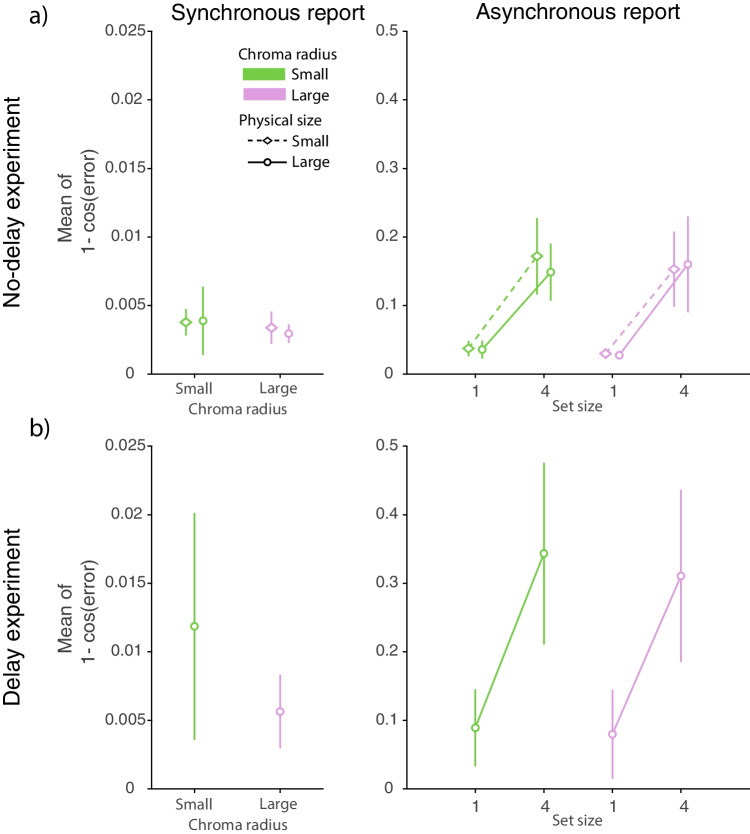
Summary statistics for the analogue report data. *Note.* Mean cosine dissimilarity for synchronous and asynchronous report conditions with different chroma radii and physical sizes of the colour wheel. **(a)** No-delay experiment. **(b)** Delay experiment. *Coloured symbols with error bars* show the mean and 95% credible intervals. A 95% credible interval is a range of parameter values that contains 95% of the posterior distribution. Given the observed data and a uniform prior, this interval represents the range of values within which the true mean has a 95% probability of falling

**Fig. 4:**
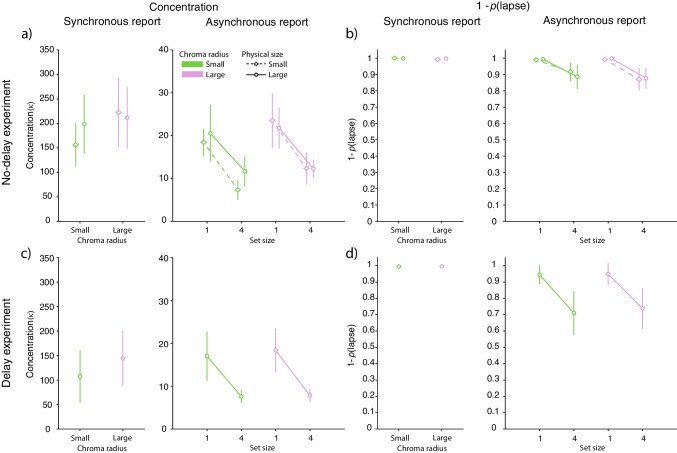
Contamination model fit for the analogue report data. *Note.* Estimates of the model parameters for synchronous and asynchronous report conditions with different chroma radii and physical sizes of the colour wheel. **(a)** Estimated concentration parameters in the no-delay experiment. **(b)** Complement of the lapse parameter estimates in the no-delay experiment.**(c)** Estimated concentration parameters in the delay experiment. **(d)** Complement of the lapse parameter estimates in the delay experiment. *Coloured symbols with error bars* show the mean and 95% credible intervals

Figure 2 shows response error distributions from the analogue report task and fits of the best fitting contamination model. Summary statistics for those distributions, calculated as cosine dissimilarity, are shown in Fig. 3. Parameters of the best fitting contamination model are shown in Fig. 4.

### Set size

We evaluate the effect of set size on performance in the analogue report task by analyzing reproduction errors on trials where observers viewed one or four coloured discs and then asynchronously reported colour of a single cued disc. We found the cosine dissimilarity increased with set size in the no-delay ($\textrm{BF}_{incl} = 2.24 \times 10^{12}$; Fig. 3a) and delay experiment ($\textrm{BF}_{incl} = 3.79 \times 10^{5}$; Fig. 3b). Importantly, this effect did not depend on the chroma radius as indicated by weak (delay experiment: $\textrm{BF}_{incl} = 0.46$) to moderate (no-delay experiment: $\textrm{BF}_{incl} = 0.13$) evidence against an interaction of set size and chroma radius. Similarly, we found moderate evidence against an interaction of set size and the physical size of the colour wheel (no-delay experiment: $\textrm{BF}_{incl} = 0.14$), as well as extreme evidence against an interaction of set size, chroma radius and the physical size of the colour wheel (no-delay experiment: $\textrm{BF}_{incl} = 0.01$). This initial analysis demonstrates the hallmark feature of human memory that the error with which objects are stored in memory increases with the number of stored objects (Bays & Husain, 2008; Palmer, 1990; Wilken & Ma, 2004).

Expectedly, the set size effect was found when the contamination model parameters were analysed instead of cosine dissimilarity (Fig. 4). The concentration parameter of the normal component was found to decrease with set size (no-delay experiment: $\textrm{BF}_{incl} = 1.27 \times 10^{8}$; delay experiment: $\textrm{BF}_{incl} = 1.19 \times 10^{4}$) and the lapse frequency was found to increase with set size (no-delay experiment: $\textrm{BF}_{incl} = 7.47 \times 10^{6}$; delay experiment: $\textrm{BF}_{incl} = 3.63 \times 10^{4}$). Similar to the analysis of the cosine dissimilarity, the set size effect on the concentration parameter was not modulated by the chroma radius (no-delay experiment: $\textrm{BF}_{incl} = 1.45$; delay experiment: $\textrm{BF}_{incl} = 0.39$), the physical size of the colour wheel (no-delay experiment: $\textrm{BF}_{incl} = 0.29$), or an interaction of chroma radius and physical size of the colour wheel (no-delay experiment: $\textrm{BF}_{incl} = 0.08$). We likewise found evidence for the robustness of the set size effect on the lapse frequency parameter, indicating that the frequency of lapses does not vary with the chroma radius (no-delay experiment: $\textrm{BF}_{incl} = 0.25$; delay experiment: $\textrm{BF}_{incl} = 0.4$), the physical size of the colour wheel (no-delay experiment: $\textrm{BF}_{incl} = 0.19$), or an interaction of chroma radius and physical size of the colour wheel (no-delay experiment: $\textrm{BF}_{incl} = 0.017$). In summary, the analysis of the cosine dissimilarity and parameters of the contamination model provides compelling evidence of an increase in error with set size in the analogue report task. Importantly the set size effect was found in both experiments and was not modulated by other factors, such as chroma radius or the physical size of the colour wheel, confirming the widely documented robustness of the set size effect on response precision in the analogue report task.

### Information maintenance

In order to investigate the role of information maintenance on the variability of response errors in the analogue report task, we first compared the error across the timing conditions (i.e. synchronous and asynchronous reproduction) in the case of set size one, followed by the comparison of asynchronous no-delay (i.e. 0 ms) and delayed (i.e. 1000 ms) reproduction.

Overall error variability, as assessed by cosine dissimilarity, was found to be larger on the asynchronous reproduction trials compared to the synchronous reproduction trials, both in the no-delay ($\textrm{BF}_{incl} = 1.69 \times 10^{14}$) and delay experiment ($\textrm{BF}_{incl} = 230.7$) (Fig. 3a & b). This result shows that observers reproduced a single colour more precisely when it remained visible, compared to when it had to be maintained in memory. Furthermore, this benefit of synchronously viewing and reproducing the stimulus was comparable across colour wheels of different physical sizes and different chroma radii as indicated by moderate evidence against an interaction of the timing conditions and colour wheel’s physical size (no-delay experiment: $\textrm{BF}_{incl} = 0.18$), weak evidence against an interaction of the timing conditions and colour wheel’s chroma radius (no-delay experiment: $\textrm{BF}_{incl} = 1.08$; delay experiment: $\textrm{BF}_{incl} = 0.38$), and strong evidence against an interaction of the timing conditions, physical size of the colour wheel and colour wheel’s chroma radius (no-delay experiment:$\textrm{BF}_{incl} = 0.04$). Together, these results show that committing an object to memory increases the error with which that object can be recovered, and that this cannot be attenuated by other factors such as the chroma or physical size of the colour wheel.

To further elucidate the effects of information maintenance on error in the analogue report task, we analysed the contamination model parameters (Fig. 4). We found extreme evidence for a difference in the concentration parameter between the synchronous and one item asynchronous reproduction (no-delay experiment: $\textrm{BF}_{incl} = 1.4 \times 10^{14}$; delay experiment: $\textrm{BF}_{incl} = 48511$) with estimated concentrations being on average larger when a colour of a visible stimulus was reproduced. Analysis of the lapse frequency revealed moderate evidence against a difference between the synchronous and no-delay asynchronous reproduction (no-delay experiment: $\textrm{BF}_{incl} = 0.28$) and moderate evidence for a difference between the synchronous and 1-s delayed asynchronous reproduction (delay experiment: $\textrm{BF}_{incl} = 3.65$), with lapses occurring more frequently on the asynchronous reproduction trials. Finally, the effect of information maintenance on the contamination model parameters did not depend on colour wheels’ chroma radii (no-delay experiment: concentration: $\textrm{BF}_{incl} = 0.61$; lapses: $\textrm{BF}_{incl} = 0.14$; delay experiment: concentration: $\textrm{BF}_{incl} = 0.82$; lapses: $\textrm{BF}_{incl} = 0.3$), or the physical size of the colour wheel (no-delay experiment: concentration $\textrm{BF}_{incl} = 0.21$; lapses $\textrm{BF}_{incl} = 0.1$), or an interaction of the colour wheels’ chroma radii and their physical sizes (no-delay experiment: concentration $\textrm{BF}_{incl} = 0.06$; lapses $\textrm{BF}_{incl} = 0.02$). In summary, the analyses so far provide evidence that errors in colour reproduction increase when items are probed immediately after stimulus disappearance (no-delay experiment) or 1 s later (delay experiment), compared to the reproduction of a still visible stimulus.

Next, we focus on the comparison of response errors obtained with asynchronous reproduction with different delays, specifically the no-delay and delay conditions (Fig. 3). This analysis revealed strong evidence for a difference in cosine dissimilarity ($\textrm{BF}_{incl} = 19.59$) with recall variability increasing between the 0-ms and 1000-ms delay interval. Additionally, the effect of delay was found to increase with set size ($\textrm{BF}_{incl} = 24.52$), with representations deteriorating faster with set size four compared to set size one. Finally, the effect of delay ($\textrm{BF}_{incl} = 0.24$) or the effect of set size on memory deterioration with delay ($\textrm{BF}_{incl} = 0.17$) did not vary with the colour wheel chroma radii, as indicated by moderate evidence against these interactions. For comparison, the magnitude of change introduced with a 1-s delay exceeded the difference observed between the synchronous and asynchronous reproduction reported in the previous analysis.

Finally, the comparison of the contamination model parameters across delays (Fig. 4) corroborated findings obtained when analysing the cosine dissimilarity. We found moderate evidence for a difference in the lapse frequency between the two delay conditions (Fig. 4b & d), with lapses increasing with delay interval ($\textrm{BF}_{incl} = 5.25$). Similar to the pattern of differences obtained for cosine dissimilarity, lapse frequency increased with delay more rapidly with larger set size ($\textrm{BF}_{incl} = 7.34$), although this effect again did not depend on the colour wheel radii (interaction of delay and colour wheel radii $\textrm{BF}_{incl} = 0.2$; interaction of delay, set size and colour wheel radii $\textrm{BF}_{incl} = 0.1$). In addition, we found weak evidence against a difference in the concentration parameters between different delay intervals ($\textrm{BF}_{incl} = 0.56$; Fig. 4a & c), an interaction of delay intervals and set size ($\textrm{BF}_{incl} = 0.39$), and strong evidence against an interaction of delay interval, set size and colour wheel radii ($\textrm{BF}_{incl} = 0.019$). Together, these results extend our initial analysis in showing that after the stimulus disappears, its representation continues to deteriorate gradually, even over a timescale of 1s, resulting in a robust increase of response error on the colour wheel.

### Colour wheel chroma radius

We next focus on investigating the effects of altering the colour space on report errors in the analogue report task. To this end, in both experiments we sampled stimuli from colour spaces of the same hues but with different chroma radii ($r_{small}$ = 25 and $r_{large}$ = 50) and asked observers to reproduce cued stimuli on the corresponding colour wheel. Despite substantially changing the feature space, in the no-delay experiment we found weak to moderate evidence against a difference in the cosine dissimilarity between colour wheels with different chroma radii on the synchronous reproduction trials ($\textrm{BF}_{incl} = 0.34$) and moderate evidence against a difference on the asynchronous reproduction trials ($\textrm{BF}_{incl} = 0.12$). The overall pattern of differences in the no-delay experiment was in agreement with that in the delay experiment. Specifically, in the delay experiment, we observed weak evidence against a difference between colour wheels’ chroma radii in the synchronous reproduction condition ($\textrm{BF}_{10} = 0.96$) and weak to moderate evidence against a difference in the asynchronous reproduction condition ($\textrm{BF}_{incl} = 0.34$). This initial analysis suggests that colour estimates were unaffected by changes in the colour space.

Analysis of the contamination model parameters confirmed these results (Fig. 4). In particular, in the no-delay experiment there was no evidence for the chroma radius to affect the concentration parameter (synchronous: $\textrm{BF}_{incl} = 0.86$; asynchronous: $\textrm{BF}_{incl} = 1.4$) or the lapse frequency (synchronous: $\textrm{BF}_{incl} = 1.2$; asynchronous: $\textrm{BF}_{incl} = 0.17$). Similarly, in the delay experiment, the concentration parameters (synchronous: $\textrm{BF}_{10} = 0.75$; asynchronous: $\textrm{BF}_{incl} = 0.37$) and the lapse frequency (synchronous: $\textrm{BF}_{10} = 0.31$; asynchronous:$\textrm{BF}_{incl} = 0.28$) were found to be comparable across colour wheels of different chroma radii. Together, analysis of cosine dissimilarity and the contamination model parameters showed that, despite substantial changes to the colour wheel’s appearance and reduced colour distinctiveness, human reproduction errors remained surprisingly comparable to those observed with a more typical colour radius.

### Motor error

We investigated the role of motor noise on reproduction errors in the analogue report task by asking observers to identify presented stimuli on colour wheels that were identical except for their physical size. Changes in the physical size of the colour wheel are not expected to influence the amplitude of motor noise, which will remain constant with respect to physical movements of the hand and consequently with respect to movements of the cursor, measured in pixels on the display. However, when measured in terms of angular error, the same amplitude of noise in cursor movement has a larger impact on a small colour wheel than a large one. A useful analogy is trying to pass a thread through the eye of a needle - constant noise (e.g. shaky hands) will more often result in missing a small eye compared to a larger eye. Similarly, in a colour reproduction task, motor noise will produce a greater dispersion of reported colours when the colours are spatially more densely packed on a smaller colour wheel. Contrary to this prediction, we found moderate evidence against a difference in cosine dissimilarity obtained on colour wheels of different physical sizes, both in the synchronous ($\textrm{BF}_{incl} = 0.23$) and asynchronous reproduction conditions ($\textrm{BF}_{incl} = 0.12$), suggesting no contribution of motor noise to overall reproduction variability in the analogue report task.

**Fig. 5:**
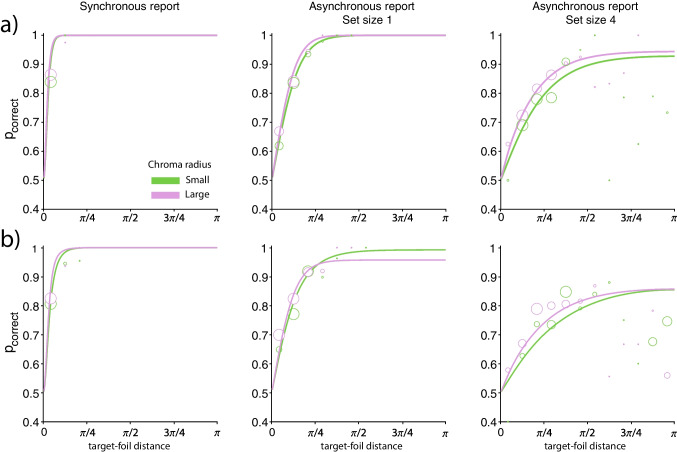
Results and psychometric function fits for the 2-AFC task. *Note.* *Coloured circles* show average proportion of correct discriminations as a function of target-foil similarity. Data is binned into 12 linearly spaced bins. The size of each circle indicates the relative proportion of trials within its respective bin compared to the total number of trials. *Solid lines* show the mean fit across observers for the multivariate normal (synchronous report) and contamination model (asynchronous report). Note that the fitting procedure used raw response data and not the binned data shown here. **(a)** No-delay experiment. **(b)** Delay experiment

These results were replicated when instead of cosine dissimilarity, we analysed the parameters of the contamination model (Fig. 4). In the synchronous reproduction condition, we observed moderate evidence against a difference in the concentration parameter of the normal component ($\textrm{BF}_{incl} = 0.29$) and weak evidence against a difference in the lapse frequency parameter ($\textrm{BF}_{incl} = 0.54$) across colour wheels of different physical sizes. Similarly, in the asynchronous condition, we found moderate evidence against a difference in the concentration parameter ($\textrm{BF}_{incl} = 0.29$) and the lapse frequency parameter ($\textrm{BF}_{incl} = 0.14$) between different physical sizes of the colour wheel, indicating that neither the precision of the normal component or the frequency of lapses is affected by motor noise.

**Fig. 6:**
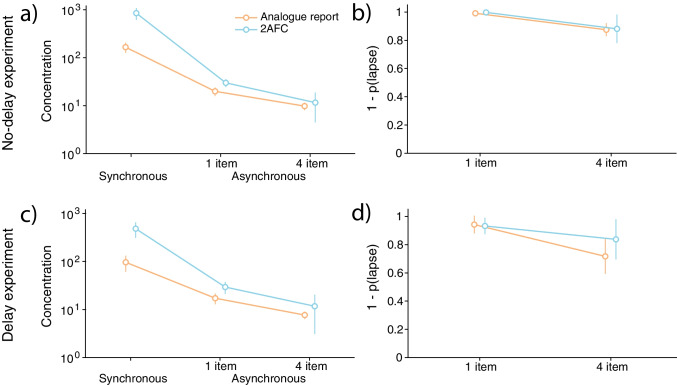
Comparison of the analogue report and 2-AFC data. *Note.* Concentration parameters plotted on log scale for ease of visualisation, and complement of lapse parameters estimated in the analogue report and 2-AFC task. **(a & b)** No-delay experiment. **(c & d)** Delay experiment. *Coloured circles with error bars* show the mean and 95% credible intervals

Comparable performance across colour wheels of different physical sizes cannot be explained by observers spending more time selecting their response in one of the two conditions. Indeed, a comparison of median response times revealed moderate evidence against a difference between the two colour wheels in the synchronous condition ($\textrm{BF}_{incl} = 0.23$) and weak evidence for a difference in the asynchronous condition ($\textrm{BF}_{incl} = 2$) where observers, contrary to what would have to be done to annul effects of motor noise, spent marginally more time (mean$\Delta$ = 275 ms) responding on the physically large colour wheel.

To put a quantitative bound on the contribution of motor noise in the analogue report task we estimated motor error variance separately for each participant and each condition (see Methods). We observed mean $\mathrm {\sigma }^{2}_{motor} = 0.2415$, corresponding to a standard deviation of $1.38^{\circ }$ on the physically large colour wheel and $4.61^{\circ }$ on the physically small colour wheel. On a more typical 8.2 dva wheel, as used by Zhang and Luck (2008) and many of the studies that followed it, the motor noise contribution to error would have s.d. of $1.69^{\circ }$.

### Decision criterion

In addition to the sources of error considered so far, another factor contributing to the overall variability in response errors might be a decision criterion related to feature adjustment. In particular, in the analogue report task observers can choose how finely to compare the features in the response space (e.g. on the colour wheel) to the one that is internally represented, and the precision of their responses will be affected by this choice. To investigate whether this freedom in deciding when to terminate the adjustment process has a beneficial or detrimental effect on performance, we directly compared the analogue report task to the 2-AFC task in which the coarseness of the required discrimination (i.e. target-foil similarity) is controlled by the experimenter.

To this end, we fitted a normal distribution to response errors and forced-choice judgments in the synchronous response conditions of the analogue report and 2-AFC task, respectively (see the Analysis section). Similarly, in the asynchronous response conditions, we fitted the two-component contamination model (von Mises + lapses) to responses from the two tasks. Figure 5 shows the 2-AFC data and the best fitting psychometric functions. Because our previous analyses did not reveal any differences between conditions with different colour wheel radii or physical sizes, we pooled the data for each observer across these conditions. Finally, we compared the concentration parameters of the normal component between the two response methods and between the synchronous and asynchronous response conditions.

In the no-delay experiment (Fig. 6a), we found extreme evidence for a difference in concentration estimates between the two tasks ($\textrm{BF}_{incl} = 2.13 \times 10^{9}$) with concentrations on average being larger in the 2-AFC task, and extreme evidence for a difference between the synchronous and asynchronous response conditions ($\textrm{BF}_{incl} = \infty$) arising from the concentration estimates decreasing when observers reported objects asynchronously, even more so with larger set size, compared to the synchronous condition. We also found extreme evidence for an interaction of these factors ($\textrm{BF}_{incl} = 8.75 \times 10^{7}$). An interaction consisted of lower concentration estimates for the analogue report task for both the synchronous ($\textrm{BF}_{10} = 175.61$) and asynchronous condition with one item ($\textrm{BF}_{10} = 8.58$), and comparable estimates across tasks in the case of the four items asynchronous condition ($\textrm{BF}_{10} = 0.34$). These results demonstrate that estimates of the contamination model’s concentration parameters are generally larger for 2-AFC data compared to continuous error data. This difference diminishes as performance decreases, eventually disappearing altogether.

Conducting the same analysis in the delay experiment (Fig. 6c) produced consistent outcomes. We again found extreme evidence for a difference in performance between the analogue report and 2-AFC task ($\textrm{BF}_{incl} = 1.16 \times 10^{6}$) indicating higher concentration estimates in the 2-AFC task. We also observed extreme evidence for a difference between the synchronous and asynchronous conditions ($\textrm{BF}_{incl} = 1.34 \times 10^{10}$) with representational precision being superior on average in the synchronous condition. Finally, we found extreme evidence for an interaction of these factors ($\textrm{BF}_{incl} = 1.36 \times 10^{5}$). Critically, comparing the two tasks within each recall condition revealed strong to moderate evidence for an advantage of the 2-AFC task in the synchronous ($\textrm{BF}_{10} = 54.58$) and asynchronous condition with one item ($\textrm{BF}_{10} = 4.88$), respectively, whereas there was weak evidence for lack of difference in the asynchronous condition with four items ($\textrm{BF}_{10} = 0.46$).

For completeness, we tested differences between the two tasks in the lapse frequency parameter obtained by fitting the two-component contamination model to response errors in the asynchronous response conditions (Fig. 6b & d). Fitting the contamination model in the two tasks returned consistent estimates of lapse rates, which increased with set size but did not differ between the tasks. Consistent with that, we found weak evidence for a difference between the two tasks (no-delay experiment: $\textrm{BF}_{incl} = 0.29$; delay experiment: $\textrm{BF}_{incl} = 0.6$), and extreme evidence for a difference between two set sizes (no-delay experiment: $\textrm{BF}_{incl} = 555$; delay experiment: $\textrm{BF}_{incl} = 14.6$ ), with performance expectedly being worse with larger set size. We also found weak evidence against an interaction between task and set size ($\textrm{BF}_{incl} = 0.37$), corroborating that lapse rates decreased consistently with set size across the two tasks. The absence of interaction was further confirmed by conducting paired *t* tests between the two tasks for each set size separately and finding no evidence for a difference with set size one (no-delay experiment: $\textrm{BF}_{10} = 0.75$; delay experiment: $\textrm{BF}_{10} = 0.33$) or set size four (no-delay experiment: $\textrm{BF}_{10} = 0.31$; delay experiment: $\textrm{BF}_{10} = 0.55$).

## Discussion

The present work explored sources of variability in response errors in the analogue report task. In two studies, we varied information processing requirements (set size, target-cue synchrony and delay) and characteristics of the stimulus space (chroma radius) and response (colour wheel radius) to test the contribution of each factor to response error. We found that human response errors are sensitive to changes in set size and maintenance requirements. In contrast, performance was unaffected by large changes in the chroma radius or physical size of the colour wheel, indicating robustness of this task to changes in the feature space, and constraining the possible contribution of motor noise to response error. Finally, comparing response variability in the analogue report task with performance in a forced-choice discrimination task, we found evidence for a limited role of adjustment criterion affecting colour identification under conditions where other sources of noise were minimized.

Changes in set size had the largest influence on reproduction variability in our experiments. We observed that performance, whether assessed as overall error variability or separate parameters of the contamination model, worsened as set size increased. Importantly, the set size effect was found in both no-delay and delay experiments with asynchronous reproduction. The results of the delay experiment, whose design closely follows a typical visual working memory experiment, are consistent with a large body of research showing that the precision with which objects are stored in memory decreases with the number of stored objects (Bays et al., 2024; Luck & Vogel, 2013; Ma et al., 2014). The no-delay experiment, where the target is cued immediately after the stimuli disappear, could in principle engage sensory memory to some extent in addition to working memory capacity (Sperling, 1960), although the cue annulus may also have acted as a meta-contrast mask, attenuating or effectively erasing sensory representations (Breitmeyer & Ogmen, 2006). In either case, the results of the no-delay experiment align with previous research in demonstrating the existence of a strong set size effect in working memory with little or even zero delay interval between stimulus and cue (Pratte, 2020; Tomić & Bays, 2024a; Tsubomi et al., 2013). This supports the view that set size differences arise at the time of encoding into working memory, while leaving open the possibility that the effect is further amplified during the course of maintenance (see below).

Previous studies have found evidence that working memories degrade over a delay (Rademaker et al., 2018; Schneegans & Bays, 2018; Shin et al., 2017). In the current study, we directly compared human reproduction variability in trials on which observers were asked to match on the colour wheel a colour that was still visible on the display (synchronous reproduction) with trials where an object was cued for reproduction once it had disappeared (asynchronous reproduction). Despite having to represent only a single item, we found convincing evidence that observers’ reproduction variability increased once the visible stimulus is removed. This was true when observers had to keep the representation in memory for 1 s (delay experiment), but also when responding was allowed immediately after the stimulus disappeared (no-delay experiment). Importantly, when comparing asynchronous reproduction with different delays, we found representations continued to deteriorate gradually, even over a timescale of 1 s, resulting in a robust increase of response errors on the colour wheel.

The majority of research involving analogue report tasks has used colour defined in CIE Lab space as a stimulus feature. This choice is motivated in part by claims about the perceptual homogeneity of CIE Lab space, such that equal physical distances between colours correspond to equal perceptual distances and discriminability. This allows researchers to select a circle varying in hue from the colour space with the expectation that the resulting space will be homogeneous. However, the same hues on a colour circle of a smaller radius are closer in CIE Lab space and should therefore become less perceptually discriminable. Here, we investigated how changes in chroma radius (i.e. colour saturation) affect reproduction precision in the analogue report task. In both experiments we sampled stimuli from colour circles with very different chroma radii, and asked observers to reproduce cued stimuli on the corresponding colour wheel. Surprisingly, in both experiments, we found that variability measured in terms of angular error did not change despite substantial differences in the colour wheel’s appearance. This held true even for the perceptual (synchronous) tasks. Although strikingly opposed to the often-cited perceptual uniformity of CIE Lab space, these results are consistent with previous work demonstrating that the saturation of monochromatic light can be substantially reduced over much of the spectrum without impairing hue discriminability (Mollon & Estévez, 1988; Tyndall, 1933). While decreasing the chroma radius even further would inevitably lead to uniform responses relative to the target stimulus (i.e. with r $\rightarrow$ 0), based on the evidence presented here, colour wheels with quite large differences in chroma radii can be expected to produce similar measurements of variability in hue estimation in analogue report tasks.

In addition to the non-uniformity along the chroma axis demonstrated here, previous studies have found evidence for non-uniformity of CIE Lab in hue space (Bae et al., 2014; Panichello et al., 2019). This is observed as biases and changes in response variability for stimuli at different points on the colour wheel. It is possible that changes in chroma could either attenuate or intensify these variations. In particular, previous work has demonstrated that colour experience of hue and chroma is not organized into independent psychological dimensions (Burns & Shepp, 1988), and a long line of research has argued that higher cognitive processes such as short-term memory inherit properties of colour perception (Allred & Flombaum, 2014). Notably, the full geometry of colour perception is too complex to be captured by the three dimensions of Lab space, or even by any Euclidean space, making the goal of a fully uniform colour wheel unattainable (Bujack et al., 2022; Ennis & Zaidi, 2019).

Several previous studies have argued that motor noise affecting selection of a feature in analogue report tasks significantly contributes to the overall response variability. On this basis, a number of studies have explicitly incorporated motor noise components into computational models of behaviour (Schurgin et al., 2020; van den Berg et al., 2012), or offered motor noise as an explanation if models fail to capture some aspects of the empirical data (Sims, 2015). And while the important role of motor noise in the execution of visuo-motor tasks has been well documented (van Beers, 2009), the overall contribution of response noise on the analogue report task has not been directly examined until now. To investigate the extent to which noise in motor output contaminates fidelity estimates in the analogue report task, we asked observers to identify the target colour on colour wheels of different physical sizes. We hypothesized that, to the extent that motor noise introduces error into human colour reproduction, that effect should be constant across changes in colour wheel size, and should therefore contribute a larger angular error on a small colour wheel compared to a large one. In contrast, we found evidence that colour wheels of different sizes provided equivalent distributions of angular errors. These results indicate that motor noise does not meaningfully affect fidelity estimates in the analogue report task.

Recently, Sutterer et al. (2022) investigated the contribution of motor noise on working memory reproduction errors by using a delayed estimation task. Assuming that motor responses are noisier when performed by the non-dominant compared to the dominant hand, the authors asked observers to asynchronously identify one of the previously presented colours with their dominant and non-dominant hands. They reasoned that if motor noise contaminates responses in the analogue report task, they ought to see stronger contamination when observers respond with their non-dominant hand compared to the dominant hand. Their data showed evidence against such an effect, both at the level of overall variability and parameters of a descriptive model. Consistent with Sutterer et al. (2022), we found more direct evidence against an effect of motor noise during asynchronous reproduction; in addition to that study, we also found evidence against an effect of motor noise on the synchronous reproduction trials. Our findings have important implications for modelling of analogue report data. In particular, if there is negligible contribution of motor noise to overall error distribution, including a motor noise component in a model runs the risk of becoming an adjustment parameter that “mops up” unexplained variance and superficially improves the model’s fit.

In psychophysics, forced-choice procedures have historically been used more widely and often regarded as superior to methods of adjustment (Kingdom & Prins, 2016). Beyond practical reasons, which became less dominant with the advancement of computer technology, one concern was that responses in the latter task depend on a subjective decision of when to terminate the adjustment process (i.e. how similar is “similar enough”). Here we explicitly investigated the role of this adjustment criterion by comparing performances on the analogue report task with the 2-AFC task, where no equivalent decision can be made. Specifically, we compared precision of observers’ responses in the analogue report task with the precision parameters of a psychometric function fitted to the 2-AFC data. Across two experiments, we found evidence for a difference in estimated variability between the 2-AFC task and the analogue report task, with variability being on average smaller in the 2-AFC task. Importantly, however, this superior performance in 2-AFC was limited to conditions where representational noise was relatively low, i.e. in the synchronous and 1-item asynchronous conditions. This suggests that the contribution of variability in the decision criterion to overall error is small compared to other sources of variability. Indeed, when observers asynchronously reported one of four items, performance in the two tasks was indistinguishable, suggesting that internal noise arising from sharing of limited mnemonic resources overpowered adjustment noise and obscured its effect.

It is possible that 2-AFC and analogue report tasks differ in other aspects beyond decision noise. Specifically, the two tasks may engage distinct top-down strategies of encoding and maintaining visual information. While the existence of the decision component in continuous report is indisputable, any discussion of possible differences in strategy between the two tasks would be more speculative. In order to account for the observed pattern of psychophysical differences between the two tasks, any ostensible difference in strategy would need to have effects that diminish with manipulations of set size and delay that increase representational noise. Because of that, we find it more likely that the differences between the two tasks arise from a constant factor whose contribution to overall error is small compared to other sources, aligning with the theoretical concept of decision noise, rather than a complex interplay of strategies that might masquerade as such a factor. Crucially, our main conclusion is methodological in nature and remains justified irrespective of any top-down differences between the tasks: precision estimates from these two methods may exhibit discrepancies when the effect of representational noise is small (e.g. low set size), but they converge when the effects of representational noise become more prominent (e.g. high set size).

The question of different top-down strategies prompted a recent study conducted by Cohen-Dallal et al. (2022), who investigated the role of expectations about an upcoming response method on performance. To this end, they randomly interleaved trials from the analogue report and change detection task, which were otherwise identical until the response stage. They found that when observers expected the continuous reproduction of orientation, manipulated by having more analogue report relative to change detection trials within a testing session, reproduction precision increased compared to sessions in which the prevailing trial type was change detection. In contrast, they did not find that expectation of a change detection trial affected detection accuracy. It is important to note that, compared to our study, Cohen-Dallal et al. (2022) did not directly compare performance across the two tasks. Rather, they focused on how expectations about the upcoming trials affect memory formation and maintenance within each task separately. This study therefore demonstrates that people engage in different encoding and maintenance strategies across the two tasks when an upcoming response method is somewhat but not fully predictable.

Besides rigorous quantitative comparison of the analogue report and forced-choice methods employed in our study, a less formal but still relevant test of methods’ comparability is based on the ability to replicate effects across different methods. Recently, Hu et al. (2023) examined the replicability of several phenomena demonstrated using discrete response methods by using the analogue report task. In particular, they identified the recency effect with the sequential presentation, prioritization of items within an array, and distractor effects (i.e. suffix interference) in visual working memory as basic phenomena of interest. Across two experiments using analogue report of orientation, they replicated all main findings previously observed with discrete response methods: better recall of the most recently presented item, better recall of prioritized items compared to other items, and a disruptive effect of a distracting object. This study adds to evidence accumulated over recent years on the replicability of classical psychophysical findings with the analogue report task (Bays et al., 2024; Luck & Vogel, 2013; Oberauer et al., 2018), suggesting the convergence of analogue report and discrete response methods.

Given the widespread popularity of using colour as a stimulus feature in the analogue report task, we also chose to focus on it in the current study. Some of the findings presented here, such as the effect of set size and information maintenance, have been previously demonstrated for other visual features (e.g. Tomić and Bays 2024b; also see Bays et al. 2024 for a review). In fact, the analogue report task has been extensively employed with various features, including orientation, angular location, motion direction, spatial frequency, abstract shapes, and can in principle be used with any other feature that allows for fine adjustments. While some of the results presented here are inherently tied to colour as a feature (e.g. the effects of saturation), most of our observations (e.g. roles of motor error and decision criterion) are expected to be general across other stimulus features employed in the analogue report task.

Similarly, in the present study, observers used a computer mouse to select the remembered colour, however, other studies employing continuous response tasks utilized different methods to collect responses, such as response dial adjustments (e.g. Schneegans & Bays, 2017), keyboard presses (e.g. van den Berg et al., 2012), ballistic finger movements or “swipes” (e.g. Schneegans and Bays 2016; Tomić and Bays 2024a), trackball adjustments (e.g. Töpfer et al., 2022). Because these different response methods require distinct motor actions, they could theoretically lead to varying levels of response variability (Menozzi et al., 2016). To the extent that observations using these methods largely mirror those made using mouse clicks, our current findings can be expected to generalize to them, however we leave systematic comparison of response methods for a future study.

## Supplementary Information

Below is the link to the electronic supplementary material.

Supplementary file 1 (pdf 301 KB)

## Data Availability

Data and analysis code are publicly available at http://doi.org/10.17863/CAM.100693.

## Declarations

### Open practices statement

Data and analysis code are publicly available at http://doi.org/10.17863/CAM.100693. None of the experiments was preregistered.

## References

[CR1] Allred, S. R., & Flombaum, J. I. (2014). Relating color working memory and color perception. *Trends in Cognitive Sciences,**18*(11), 562–565. 10.1016/j.tics.2014.06.002 25038028 10.1016/j.tics.2014.06.002

[CR2] Alvarez, G. A., & Cavanagh, P. (2008). Visual short-term memory operates more efficiently on boundary features than on surface features. *Perception & Psychophysics,**70*(2), 346–364. 10.3758/PP.70.2.346 18372755 10.3758/pp.70.2.346 PMC2621012

[CR3] Bae, G.-Y., Olkkonen, M., Allred, S. R., Wilson, C., & Flombaum, J. I. (2014). Stimulus-specific variability in color working memory with delayed estimation. *Journal of Vision,**14*(4), 7–7. 10.1167/14.4.7 24715329 10.1167/14.4.7

[CR4] Bays, P. M. (2014). Noise in neural populations accounts for errors in working memory. *Journal of Neuroscience,**34*(10), 3632–3645. 10.1523/JNEUROSCI.3204-13.2014 24599462 10.1523/JNEUROSCI.3204-13.2014 PMC3942580

[CR5] Bays, P. M. (2016). A signature of neural coding at human perceptual limits. *Journal of Vision,**16*(11), 4. 10.1167/16.11.4 27604067 10.1167/16.11.4 PMC5024667

[CR6] Bays, P. M., Catalao, R. F. G., & Husain, M. (2009). The precision of visual working memory is set by allocation of a shared resource. *Journal of Vision,**9*, 1–11. 10.1167/9.10.7 19810788 10.1167/9.10.7 PMC3118422

[CR7] Bays, P. M., & Husain, M. (2008). Dynamic shifts of limited working memory resources in human vision. *Science,**321*(5890), 851–854. 10.1126/science.1158023 18687968 10.1126/science.1158023 PMC2532743

[CR8] Bays, P. M., Schneegans, S., Ma, W. J., & Brady, T. F. (2024). Representation and computation in visual working memory. *Nature Human Behaviour,**8*, 1016–1034. 10.1038/s41562-024-01871-2 10.1038/s41562-024-01871-2 38849647

[CR9] Brady, T. F., Konkle, T., Gill, J., Oliva, A., & Alvarez, G. A. (2013). Visual Long-Term Memory Has the Same Limit on Fidelity as Visual Working Memory. *Psychological Science,**24*(6), 981–990. 10.1177/0956797612465439 23630219 10.1177/0956797612465439

[CR10] Brainard, D. H. (1997). The psychophysics toolbox. *Spatial Vision,**10*(4), 433–436. 10.1163/156856897X00357 9176952

[CR11] Breitmeyer, B. G., & Ogmen, H. (2006). *Visual masking: Time slices through conscious and unconscious vision*. Oxford University Press.

[CR12] Bujack, R., Teti, E., Miller, J., Caffrey, E., & Turton, T. L. (2022). The non-riemannian nature of perceptual color space. *Proceedings of the National Academy of Sciences,**119*(18), e2119753119. 10.1073/pnas.2119753119 10.1073/pnas.2119753119 PMC9170152 35486695

[CR13] Burns, B., & Shepp, B. E. (1988). Dimensional interactions and the structure of psychological space: The representation of hue, saturation, and brightness. *Perception & Psychophysics,**43*(5), 494–507. 10.3758/BF03207885 3380640 10.3758/bf03207885

[CR14] Cardozo, B. L. (1965). Adjusting the Method of Adjustment: SD vs DL. *The Journal of the Acoustical Society of America,**37*(5), 786–792. 10.1121/1.1909439

[CR15] Cohen-Dallal, H., Markus, O., & Pertzov, Y. (2022). Adaptive visual working memory: Expecting a delayed estimation task enhances visual working memory precision. *Journal of Experimental Psychology: Human Perception and Performance,*. 10.1037/xhp0001066 10.1037/xhp0001066 36355704

[CR16] Ennis, R. J., & Zaidi, Q. (2019). Geometrical structure of perceptual color space: Mental representations and adaptation invariance. *Journal of Vision,**19*(12), 1. 10.1167/19.12.1 31573606 10.1167/19.12.1 PMC6779095

[CR17] Faisal, A. A., Selen, L. P. J., & Wolpert, D. M. (2008). Noise in the nervous system. *Nature Reviews Neuroscience,**9*(4), 292–303. 10.1038/nrn2258 18319728 10.1038/nrn2258 PMC2631351

[CR18] Fougnie, D., Suchow, J. W., & Alvarez, G. A. (2012). Variability in the quality of visual working memory. *Nature Communications,**3*, 1229. 10.1038/ncomms2237 23187629 10.1038/ncomms2237 PMC3563332

[CR19] Green, D. M., & Swets, J. A. (1966). *Signal detection theory and psychophysics*. Wiley.

[CR20] Hinne, M., Gronau, Q. F., van den Bergh, D., & Wagenmakers, E.-J. (2020). A Conceptual Introduction to Bayesian Model Averaging. *Advances in Methods and Practices in Psychological Science,**3*(2), 200–215. 10.1177/2515245919898657

[CR21] Hu, Y., Allen, R. J., Baddeley, A. D., & Hitch, G. J. (2023). Visual working memory phenomena based on categorical tasks replicate using a continuous measure: A simple interpretation and some methodological considerations. *Attention, Perception, & Psychophysics*. 10.3758/s13414-023-02656-x 10.3758/s13414-023-02656-x PMC10372120 36754918

[CR22] JASP Team. (2022). JASP (Version 0.16.1)[Computer software]. https://jasp-stats.org/

[CR23] Keysers, C., Gazzola, V., & Wagenmakers, E.-J. (2020). Using bayes factor hypothesis testing in neuroscience to establish evidence of absence. *Nature Neuroscience,**23*(7), 788–799. 10.1038/s41593-020-0660-4 32601411 10.1038/s41593-020-0660-4 PMC7610527

[CR24] Kingdom, F. A. A., & Prins, N. (2016). *Psychophysics: A practical introduction* (Second edition). Elsevier/Academic Press.

[CR25] Kontsevich, L. L., & Tyler, C. W. (1999). Bayesian adaptive estimation of psychometric slope and threshold. *Vision Research,**39*(16), 2729–2737. 10492833 10.1016/s0042-6989(98)00285-5

[CR26] Lee, M. D., & Wagenmakers, E. -J. (2013). *Bayesian cognitive modeling: A practical course*. Cambridge University Press.

[CR27] Liang, F., Paulo, R., Molina, G., Clyde, M. A., & Berger, J. O. (2008). Mixtures of g Priors for Bayesian Variable Selection. *Journal of the American Statistical Association,**103*, 410–423. 10.1198/016214507000001337

[CR28] Luck, S. J., & Vogel, E. K. (2013). Visual working memory capacity: From psychophysics and neurobiology to individual differences. *Trends in Cognitive Sciences,**17*(8), 391–400. 10.1016/j.tics.2013.06.006 23850263 10.1016/j.tics.2013.06.006 PMC3729738

[CR29] Ma, W. J., Husain, M., & Bays, P. M. (2014). Changing concepts of working memory. *Nature Neuroscience,**17*(3),. 10.1038/nn.3655 10.1038/nn.3655 PMC4159388 24569831

[CR30] McMaster, J. M., Tomić, I., Schneegans, S., & Bays, P. M. (2022). Swap errors in visual working memory are fully explained by cue-feature variability. *Cognitive Psychology,**137*, 101493. 10.1016/j.cogpsych.2022.101493 35777189 10.1016/j.cogpsych.2022.101493 PMC7613075

[CR31] Menozzi, M., Huang, Y.-Y., & Abt, N. A. (2016). Accuracy of non-visual directional pointing with various manual input devices. *International Journal of Industrial Ergonomics,**53*, 258–266. 10.1016/j.ergon.2016.02.001

[CR32] Mollon, J. D., & Estévez, O. (1988). Tyndall’s paradox of hue discrimination. *Journal of the Optical Society of America A,**5*(1), 151. 10.1364/JOSAA.5.000151 10.1364/josaa.5.000151 3351652

[CR33] Murray, A. M., Nobre, A. C., Clark, I. A., Cravo, A. M., & Stokes, M. G. (2013). Attention Restores Discrete Items to Visual Short-Term Memory. *Psychological Science,**24*(4), 550–556. 10.1177/0956797612457782 23436786 10.1177/0956797612457782 PMC4138001

[CR34] Oberauer, K., Lewandowsky, S., Awh, E., Brown, G. D. A., Conway, A., Cowan, N., . . . Ward, G. (2018). Benchmarks for models of short-term and working memory. *Psychological Bulletin,**144*(9), 885–958. 10.1037/bul0000153 10.1037/bul0000153 30148379

[CR35] Palmer, J. (1990). Attentional limits on the perception and memory of visual information. *Journal of Experimental Psychology. Human Perception and Performance,**16*(2), 332–350. 2142203 10.1037//0096-1523.16.2.332

[CR36] Panichello, M. F., DePasquale, B., Pillow, J. W., & Buschman, T. J. (2019). Error-correcting dynamics in visual working memory. *Nature Communications,**10*(1),. 10.1038/s41467-019-11298-3 10.1038/s41467-019-11298-3 PMC6662698 31358740

[CR37] Pelli, D. G. (1997). The VideoToolbox software for visual psychophysics: Transforming numbers into movies. *Spatial Vision,**10*(4), 437–442. 9176953

[CR38] Pratte, M. S. (2018). Iconic Memories Die a Sudden Death. *Psychological Science,**29*(6), 877–887. 10.1177/0956797617747118 29671682 10.1177/0956797617747118 PMC5993568

[CR39] Pratte, M. S. (2020). Set size effects on working memory precision are not due to an averaging of slots. *Attention, Perception, & Psychophysics,**82*(6), 2937–2949. 10.3758/s13414-019-01902-5 10.3758/s13414-019-01902-5 PMC7387150 32350828

[CR40] Prinzmetal, W., Amiri, H., Allen, K., & Edwards, T. (1998). Phenomenology of attention: I. Color, location, orientation, and spatial frequency. *Journal of Experimental Psychology: Human Perception and Performance,**24*(1), 261–282. 10.1037/0096-1523.24.1.261

[CR41] Rademaker, R. L., Park, Y. E., Sack, A. T., & Tong, F. (2018). Evidence of gradual loss of precision for simple features and complex objects in visual working memory. *Journal of Experimental Psychology: Human Perception and Performance.*[SPACE]10.1037/xhp0000491 29494191 10.1037/xhp0000491 PMC5975117

[CR42] Richter, F. R., Cooper, R. A., Bays, P. M., & Simons, J. S. (2016). Distinct neural mechanisms underlie the success, precision, and vividness of episodic memory. *eLife,**5*, e18260. 10.7554/eLife.18260 27776631 10.7554/eLife.18260 PMC5079745

[CR43] Schneegans, S., & Bays, P. M. (2016). No fixed item limit in visuospatial working memory. *Cortex,**83*, 181–193. 10.1016/j.cortex.2016.07.021 27565636 10.1016/j.cortex.2016.07.021 PMC5043407

[CR44] Schneegans, S., & Bays, P. M. (2017). Neural architecture for feature binding in visual working memory. *The Journal of Neuroscience,**37*(14), 3913–3925. 10.1523/JNEUROSCI.3493-16.2017 28270569 10.1523/JNEUROSCI.3493-16.2017 PMC5394900

[CR45] Schneegans, S., & Bays, P. M. (2018). Drift in Neural Population Activity Causes Working Memory to Deteriorate Over Time. *The Journal of Neuroscience,**38*(21), 4859–4869. 10.1523/JNEUROSCI.3440-17.2018 29703786 10.1523/JNEUROSCI.3440-17.2018 PMC5966793

[CR46] Schurgin, M. W., Wixted, J. T., & Brady, T. F. (2020). Psychophysical scaling reveals a unified theory of visual memory strength. *Nature Human Behaviour,**4*(11), 1156–1172. 10.1038/s41562-020-00938-0 32895546 10.1038/s41562-020-00938-0

[CR47] Shin, H., Zou, Q., & Ma, W. J. (2017). The effects of delay duration on visual working memory for orientation. *Journal of Vision,**17*(14), 10. 10.1167/17.14.10 29234786 10.1167/17.14.10 PMC6097585

[CR48] Sims, C. R. (2015). The cost of misremembering: Inferring the loss function in visual working memory. *Journal of Vision,**15*(3), 2–2. 10.1167/15.3.2 25740875 10.1167/15.3.2

[CR49] Sperling, G. (1960). The information available in brief visual presentations. *Psychological Monographs: General and Applied,**74*(11), 1–29. 10.1037/h0093759

[CR50] Stefan, A. M., Gronau, Q. F., Schönbrodt, F. D., & Wagenmakers, E.-J. (2019). A tutorial on bayes factor design analysis using an informed prior. *Behavior Research Methods,**51*(3), 1042–1058. 10.3758/s13428-018-01189-8 30719688 10.3758/s13428-018-01189-8 PMC6538819

[CR51] Sutterer, D., Rosca, C. G., & Woodman, G. F. (2022). Does motor noise contaminate estimates of the precision of visual working memory? *Visual Cognition,**30*(3), 195–201. 10.1080/13506285.2022.2044947 36061238 10.1080/13506285.2022.2044947 PMC9431962

[CR52] Tang, M. F., Ford, L., Arabzadeh, E., Enns, J. T., Visser, T. A. W., & Mattingley, J. B. (2020). Neural dynamics of the attentional blink revealed by encoding orientation selectivity during rapid visual presentation. *Nature Communications,**11*(1), 434. 10.1038/s41467-019-14107-z 31974370 10.1038/s41467-019-14107-z PMC6978470

[CR53] Taylor, R., & Bays, P. M. (2018). Efficient coding in visual working memory accounts for stimulus-specific variations in recall. *The Journal of Neuroscience,* 1018–18. 10.1523/JNEUROSCI.1018-18.2018 10.1523/JNEUROSCI.1018-18.2018 PMC6083451 30006363

[CR54] Thibault, L., van den Berg, R., Cavanagh, P., & Sergent, C. (2016). Retrospective Attention Gates Discrete Conscious Access to Past Sensory Stimuli (F. P. de Lange, Ed.). *PLoS One,**11*(2), e0148504. 10.1371/journal.pone.0148504 10.1371/journal.pone.0148504 PMC4749386 26863625

[CR55] Tolhurst, D., Movshon, J., & Dean, A. (1983). The statistical reliability of signals in single neurons in cat and monkey visual cortex. *Vision Research,**23*(8), 775–785. 10.1016/0042-6989(83)90200-6 10.1016/0042-6989(83)90200-6 6623937

[CR56] Tomić, I., & Bays, P. M. (2018). Internal but not external noise frees working memory resources. *PLOS Computational Biology,**14*(10), e1006488. 10.1371/journal.pcbi.1006488 30321172 10.1371/journal.pcbi.1006488 PMC6201966

[CR57] Tomić, I., & Bays, P. M. (2024a). A dynamic neural resource model bridges sensory and working memory. *eLife,**12*, RP91034. 10.7554/eLife.91034.3 10.7554/eLife.91034 PMC11068358 38700934

[CR58] Tomić, I., & Bays, P. M. (2024b). Perceptual similarity judgments do not predict the distribution of errors in working memory. *Journal of Experimental Psychology: Learning, Memory, and Cognition,**50*(4), 535–549. 10.1037/xlm0001172 10.1037/xlm0001172 PMC7615806 36442045

[CR59] Töpfer, F. M., Barbieri, R., Sexton, C. M., Wang, X., Soch, J., Bogler, C., & Haynes, J.-D. (2022). Psychophysics and computational modeling of feature-continuous motion perception. *Journal of Vision,**22*(11), 16. 10.1167/jov.22.11.16 36306146 10.1167/jov.22.11.16 PMC9624271

[CR60] Tsubomi, H., Fukuda, K., Watanabe, K., & Vogel, E. K. (2013). Neural Limits to Representing Objects Still within View. *The Journal of Neuroscience,**33*(19), 8257–8263. 10.1523/JNEUROSCI.5348-12.2013 23658165 10.1523/JNEUROSCI.5348-12.2013 PMC4049283

[CR61] Tyndall, E. P. T. (1933). Chromaticity sensibility to wave-length difference as a function of purity*. *Journal of the Optical Society of America,**23*(1), 15. 10.1364/JOSA.23.000015

[CR62] van Beers, R. J. (2009). Motor Learning Is Optimally Tuned to the Properties of Motor Noise. *Neuron,**63*(3), 406–417. 10.1016/j.neuron.2009.06.025 19679079 10.1016/j.neuron.2009.06.025

[CR63] van den Berg, R., Shin, H., Chou, W.-C., George, R., & Ma, W. J. (2012). Variability in encoding precision accounts for visual short-term memory limitations. *Proceedings of the National Academy of Sciences,**109*(22), 8780–8785. 10.1073/pnas.1117465109 10.1073/pnas.1117465109 PMC3365149 22582168

[CR64] Wagenmakers, E.-J., Love, J., Marsman, M., Jamil, T., Ly, A., Verhagen, J., . . . Morey, R. D. (2018). Bayesian inference for psychology. Part II: Example applications with JASP. *Psychonomic Bulletin & Review,**25*(1), 58–76. 10.3758/s13423-017-1323-7 10.3758/s13423-017-1323-7 PMC5862926 28685272

[CR65] Wier, C. C., Jesteadt, W., & Green, D. M. (1976). A comparison of method-of-adjustment and forced-choice procedures in frequency discrimination. *Perception & Psychophysics,**19*(1), 75–79. 10.3758/BF03199389

[CR66] Wilken, P., & Ma, W. J. (2004). A detection theory account of change detection. *Journal of Vision,**4*(12), 11–11. 10.1167/4.12.11 10.1167/4.12.11 15669916

[CR67] Zhang, W., & Luck, S. J. (2008). Discrete fixed-resolution representations in visual working memory. *Nature,**453*(7192), 233–235. 10.1038/nature06860 18385672 10.1038/nature06860 PMC2588137

