# Supplementary material for: Dissecting the components of error in analogue report tasks
Source: Behav Res Methods. 2024 Jul 8;56(8):8196–213. doi: 10.3758/s13428-024-02453-w (PMC11525414; doi:10.3758/s13428-024-02453-w)
Supplement: Supplementary file 1 — (pdf 301 KB) [file 13428_2024_2453_MOESM1_ESM.pdf]

Dissecting the components of error in analogue report tasks

Supplementary Information

Ivan Tomić, Dagmar Adamcová, Máté Fehér, and Paul M. Bays

Supplementary Information

Figure S1

Expected Bayes factor for the null hypothesis

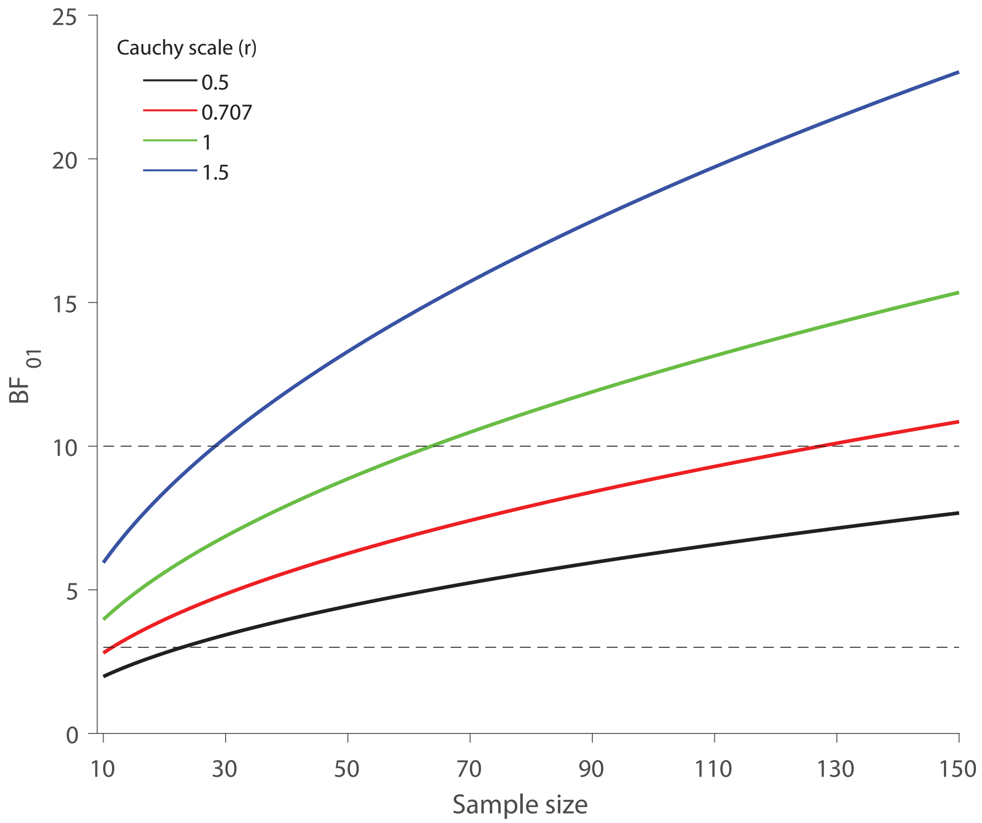

Note. *Expected Bayes factor for the null hypothesis when the null is true as a function of sample size and prior width. Dashed lines indicate BF = 3 (moderate evidence) and BF = 10 (strong evidence). Calculations are based on the equations in Rouder et al. (2009). The red line corresponds to commonly used prior.*

References

Rouder, J. N., Speckman, P. L., Sun, D., Morey, R. D., & Iverson, G. (2009). Bayesian t tests for accepting and rejecting the null hypothesis. *Psychonomic Bulletin & Review*, *16*(2), 225–237. https://doi.org/10.3758/PBR.16.2.225
